# Supplementary material for: Bacteria use exogenous peptidoglycan as a danger signal to trigger biofilm formation
Source: Nat Microbiol. 2025 Jan 3;10(1):144–57. doi: 10.1038/s41564-024-01886-5 (PMC11726461; doi:10.1038/s41564-024-01886-5)
Supplement: Supplementary file 1 — Reporting Summary [file 41564_2024_1886_MOESM1_ESM.pdf]

nature portfolio

Corresponding author(s): Knut Drescher
Last updated by author(s): Nov 8, 2024

Reporting Summary

Nature Portfolio wishes to improve the reproducibility of the work that we publish. This form provides structure for consistency and transparency in reporting. For further information on Nature Portfolio policies, see our Editorial Policies and the Editorial Policy Checklist.

Statistics

For all statistical analyses, confirm that the following items are present in the figure legend, table legend, main text, or Methods section.

n/a	Confirmed	
☐	☒	The exact sample size (*n*) for each experimental group/condition, given as a discrete number and unit of measurement
☐	☒	A statement on whether measurements were taken from distinct samples or whether the same sample was measured repeatedly
☐	☒	The statistical test(s) used AND whether they are one- or two-sided<br>*Only common tests should be described solely by name; describe more complex techniques in the Methods section.*
☒	☐	A description of all covariates tested
☐	☒	A description of any assumptions or corrections, such as tests of normality and adjustment for multiple comparisons
☐	☒	A full description of the statistical parameters including central tendency (e.g. means) or other basic estimates (e.g. regression coefficient) AND variation (e.g. standard deviation) or associated estimates of uncertainty (e.g. confidence intervals)
☐	☒	For null hypothesis testing, the test statistic (e.g. *F*, *t*, *r*) with confidence intervals, effect sizes, degrees of freedom and *P* value noted<br>*Give P values as exact values whenever suitable.*
☒	☐	For Bayesian analysis, information on the choice of priors and Markov chain Monte Carlo settings
☒	☐	For hierarchical and complex designs, identification of the appropriate level for tests and full reporting of outcomes
☒	☐	Estimates of effect sizes (e.g. Cohen's *d*, Pearson's *r*), indicating how they were calculated

Our web collection on statistics for biologists contains articles on many of the points above.

Software and code

Policy information about availability of computer code

Data collection	Nikon NIS Elements Advanced Research 4.5 software and Micro-Manager 2.0beta were used to control microscopes for image acquisition.
Data analysis	BiofilmQ v0.2.2 was used to analyse biofilm images: https://drescherlab.org/data/biofilmQ/ .<br>Bar graphs were generated using Graphpad Prism v9, which was also used for performing all statistical tests indicated in figure captions.<br>Three-dimensional rendering of confocal microscopy images of bacterial cells was performed using Paraview v5.10.1, based on segmentation results and output from BiofilmQ.<br>CLC Genomics Workbench v10.1.1 (Qiagen) was used to process RNA-seq data obtained by Illumina sequencing.<br>Source code of the Matlab script we used to quantify the 3D biofilm biovolume is available in a Github repository: https://github.com/knutdrescher/biofilm-3D-biovolume.

For manuscripts utilizing custom algorithms or software that are central to the research but not yet described in published literature, software must be made available to editors and reviewers. We strongly encourage code deposition in a community repository (e.g. GitHub). See the Nature Portfolio guidelines for submitting code & software for further information.

Data

Policy information about availability of data

All manuscripts must include a data availability statement. This statement should provide the following information, where applicable:

- Accession codes, unique identifiers, or web links for publicly available datasets
- A description of any restrictions on data availability
- For clinical datasets or third party data, please ensure that the statement adheres to our policy

Transcriptome data are available at the National Center for Biotechnology Information Gene Expression Omnibus under the accession number GSE216690 (https://www.ncbi.nlm.nih.gov/geo/query/acc.cgi?acc=GSE216690).
Image data are available on the Zenodo repository (DOI: 10.5281/zenodo.14054836).
Processed data used in this study are available as Source Data together with the manuscript.

Human research participants

Policy information about studies involving human research participants and Sex and Gender in Research.

Reporting on sex and gender	n/a
Population characteristics	n/a
Recruitment	n/a
Ethics oversight	n/a

Note that full information on the approval of the study protocol must also be provided in the manuscript.

Field-specific reporting

Please select the one below that is the best fit for your research. If you are not sure, read the appropriate sections before making your selection.

☒ Life sciences ☐ Behavioural & social sciences ☐ Ecological, evolutionary & environmental sciences

For a reference copy of the document with all sections, see nature.com/documents/nr-reporting-summary-flat.pdf

Life sciences study design

All studies must disclose on these points even when the disclosure is negative.

Sample size	The number (n) of independent replicate experiments that were performed for each experiment was determined by the minimum number of biological replicates required for good data distribution and statistics. For most experiments reported in this study n=3, which is a generally accepted number of independent replicates in the research field. However, for some assays n>3 independent replicate were acquired when the assay cold be done in higher throughput. The exact sample size for each experiment is indicated in the figure captions.
Data exclusions	No data were excluded.
Replication	Each experiment was replicated n times (and n is given in each figure for each experiment). Although the exact quantitative results differ between replicates, the qualitative results were the same, so that it is reasonable to state that the "replication was successful".
Randomization	There were many bacterial cells within each of the n replicates. Because of the large sample size for each replicate, a representative number of samples were collected for each replicate. There was no allocation of samples into experimental groups, beyond conducting independent biological replicates.
Blinding	Blinding of group allocation is irrelevant to our data analysis, because there was no allocation to experimental groups, beyond collecting n replicates, all of which were analyzed by software equally.

Reporting for specific materials, systems and methods

We require information from authors about some types of materials, experimental systems and methods used in many studies. Here, indicate whether each material, system or method listed is relevant to your study. If you are not sure if a list item applies to your research, read the appropriate section before selecting a response.

Materials & experimental systems

n/a	Involved in the study	
☒	☐	Antibodies
☒	☐	Eukaryotic cell lines
☒	☐	Palaeontology and archaeology
☒	☐	Animals and other organisms
☒	☐	Clinical data
☒	☐	Dual use research of concern

Methods

n/a	Involved in the study	
☒	☐	ChIP-seq
☒	☐	Flow cytometry
☒	☐	MRI-based neuroimaging
